# The Prevalence of Illness Anxiety Disorder Among Adults in Taif City, Saudi Arabia: A Cross-Sectional Study

**DOI:** 10.7759/cureus.55255

**Published:** 2024-02-29

**Authors:** May Abdulaziz, Taif S Alharthi, Mohammad I Alwuthaynani, Ghadah Althobaiti, Abdullah E Alsobaie, Marah Alqurashi, Riyadh Alamrai, Abdulmohsin Ahmadjee

**Affiliations:** 1 College of Medicine, Taif University, Taif, SAU; 2 Collage of Medicine, Taif University, Taif, SAU

**Keywords:** saudi arabia, cardiac manifestations, prevalence, health anxiety, hypochondriasis, illness anxiety disorder

## Abstract

Introduction

Illness Anxiety Disorder (IAD), characterized by intense fear of serious illness, has been associated with performance issues at work, frequent absences, financial burdens from medical expenses, impaired daily functioning, and the onset and recurrence of coronary heart disease. This study aimed to assess the prevalence of IAD and explore its cardiac manifestations in residents of Taif City, Saudi Arabia.

Methods

A descriptive cross-sectional study was conducted among adults in Taif City, excluding those with psychiatric illnesses. Participants completed an online self-administered questionnaire, including sociodemographic information and the validated Short Version Health Anxiety Inventory (SHAI) scale.

Results

Among 415 participants, predominantly females (60%), the study found a 25.3% prevalence of IAD. Of those with IAD, 3% were diagnosed with cardiac diseases, and 27% were hospitalized due to cardiac symptoms. Twenty-five percent exhibited normal examination results after hospitalization. Factors such as female gender (p=0.006), younger age (p=0.006), single marital status (p=0.012), and a history of hospitalization due to heart symptoms (p=0.003) were associated with higher IAD scores. Married participants had a lower risk of IAD compared to singles (OR: -2.2, 95% CI: -3.9, -0.48), while a history of hospitalization due to heart symptoms increased the risk of IAD (OR: 2.8, 95% CI: 0.94, 4.7).

Conclusion

This study revealed a substantial prevalence of IAD in Taif City. Female gender, younger age, being single, and having a history of hospitalization due to heart symptoms were identified as determinants of IAD. Healthcare providers must recognize these disorders to prevent unnecessary investigations and treatments, redirecting patients to psychiatry for more cost-effective and beneficial interventions.

## Introduction

Illness Anxiety Disorder (IAD), formerly known as hypochondriasis or health anxiety, is a primary concern involving intense worry about acquiring a severe illness, as outlined in the Diagnostic and Statistical Manual of Mental Disorders, Fifth Edition (DSM-5) fifth edition in 2013 [1]. The diagnostic criteria remained the same with the publication of the DSM-5-TR in 2022 [2]. Its prevalence remains uncertain due to its recent identification [3].

While anxiety disorders generally arise in early or middle adulthood and can worsen with age, IAD specifically tends to surface during adolescence or early adulthood, potentially worsening with maturity [4]. Often, for older individuals, health-related anxiety may focus on the fear of losing their memory [5]. Risk factors encompass significant life stress, childhood abuse, parental or personal history of serious illness, excessive worrying tendencies, and Internet overuse for health-related concerns [5]. Those affected often engage in repeated doctor visits seeking reassurance, yet many avoid medical consultations out of fear of confirming their worries [1]. Diagnosis requires persistent concerns lasting at least six months, even with reassurance after thorough medical evaluations [6]. 

It is believed that IAD is composed of three domains: first, disease conviction despite a lack of medical evidence; second, fear of having a serious sickness, which raises distress; and finally, increased focus on physiologic processes, discomfort, and physical restrictions [1]. IAD patients sometimes seek additional doctors' opinions for the same medical issue because they are unsatisfied with their negative assessments. Some think their prior physicians were either ignorant or careless about their medical health [7]. IAD is marked by heightened bodily awareness, where ordinary physical sensations are misinterpreted as signs of serious illness, impacting daily life negatively [2,8]. Excessive concern associated with IAD can strain relationships and lead to various complications, including performance issues at work, frequent absences, financial burdens from medical expenses, and impaired daily functioning [9-11]. Individuals with IAD may also experience other mental health conditions such as somatic symptom disorder, different anxiety disorders, depression, or personality issues [12].

Research suggests anxiety is a potential risk factor for the onset and recurrence of coronary heart disease (CHD). Different forms of anxiety, including social anxiety, generalized anxiety, worry, and phobic symptoms, have been linked to the emergence and recurrence of CHD. However, the prevalence of anxiety problems among diagnosed CHD patients remains unclear [13].

People with IAD can be categorized as care-seekers or care-avoiders based on their medical care utilization patterns. Studies show that 61% of IAD patients alternate between seeking and avoiding care, while 25% are consistent care-seekers, and care-avoidant cases are less common [6]. In a 2021 case report, an IAD patient seeking treatment in the US displayed symptoms like sleeping difficulties, panic attacks, ruminative worries, muscle tension, weakness, and chest discomfort despite normal medical evaluations [10]. Another study in India found that 7% of 400 outpatient attendees had IAD, often coupled with depression and anxiety [7]. A 2016 case report detailed a 73-year-old Saudi man undergoing unnecessary tests costing over $170,000 due to health anxiety, significantly impacting his life [14]. These diverse studies emphasize IAD's prevalence and its association with psychiatric conditions, which underscores the need for further research, particularly in places like Saudi Arabia. Our study in Taif City aims to explore IAD prevalence and its determinants and investigate cardiac manifestations in affected individuals.

## Materials and methods

A descriptive cross-sectional study was conducted at Taif City, Saudi Arabia, between April 2023 and July 2023, among adults aged ≥ 18 years. Participants with psychiatric illness and those who didn’t live in Taif City or refused to participate were excluded. The Raosoft Sample Size Calculator was used to determine the sample size based on a 95% confidence interval, a 5% margin of error, and a 50% population proportion. The minimum required sample size was 385.

Data collection

A pilot study involving 20 participants assessed the questionnaire's clarity and understandability. We made improvements to enhance the clarity and comprehensibility of the research. These changes included refining the language used, clarifying key concepts, and providing additional explanatory details where necessary. Data were collected through a convenient sampling technique using an online self-administered questionnaire disseminated to participants by an online Google Form through different social media platforms. The questionnaire contains twenty-four questions comprising three sections. The first section captured sociodemographic information, including age, gender, marital status, education level, occupation, and monthly income of the family. Section two assessed health anxiety independently of physical health status using the Short version of the Health Anxiety Inventory (SHAI) scale [15], composed of 18 items. It is a 4-point scale ranging from 0 “not at all or rarely” to 3 “most of the time” with a Cronbach alpha of 0.89 [16]. A score of ≥18 has been utilized and recommended by previous studies to determine significant health anxiety [17]. Section three covered questions about the history of hospital visits and cardiac manifestations.

Data analysis

The data was cleaned in an Excel sheet and imported to R software version 4.2.2 (R Foundation for Statistical Computing, Vienna, Austria). The normality of the SHAI scale was tested using a histogram and Kolmogorov-Smirnov test. Descriptive statistics were used for calculating the mean and Standard deviation for the continuous variables and frequencies with percentages for categorical variables. The one-way analysis of variance (ANOVA) and two sample t-tests were used to identify variables associated with IAD. A multiple linear regression analysis was performed to identify the predictors of IAD. The p-value of ≤ 0.05 was set as the significance level of the study.

Ethical considerations

This study has been approved by the Scientific Research Ethics Committee at Taif University (application no. 44-287). Every participant was provided with a comprehensive explanation regarding the study's objectives. Subsequently, online informed written consent was obtained from all participants, affirming their voluntary participation in the study. The ethical preference of participant withdrawal at any point was emphasized. Anonymity and confidentiality regarding participants' identities and responses were maintained.

## Results

The study involved 415 participants, of which 251 (60%) were females, 265 (64%) aged 18-24 years, 276 (67%) were singles, 293 (71%) had Bachelor’s degrees, and 166 (40%) had a family income less than 10000 riyals (Table 1).

**Table 1:** Demographic characteristics of the study participants *n (%)

Characteristic	N = 415^*^
Gender	
Female	251 (60%)
Male	164 (40%)
Age	
18-24 years	265 (64%)
25-34 years	47 (11%)
35-44 years	36 (8.7%)
45-55 years	59 (14%)
more than 55 years	8 (1.9%)
Marital status	
Single	276 (67%)
Married	135 (33%)
Divorced	4 (1.0%)
Education level	
Primary school	7 (1.7%)
High school	92 (22%)
Diploma	9 (2.2%)
Bachelor’s	293 (71%)
Post-graduate	14 (3.4%)
Occupation	
Government employee	94 (23%)
Private sector employee	8 (1.9%)
Private job	6 (1.4%)
Health care provider ( government or private)	11 (2.7%)
Student	249 (60%)
I’m not working right now	47 (11%)
Income	
Less than 10,000 riyals	166 (40%)
10,000-15000 riyals	117 (28%)
More than 15000 riyals	132 (32%)

**Figure 1:**
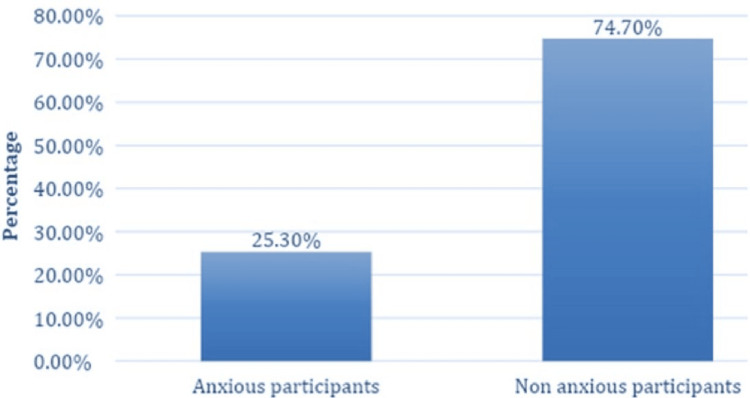
Prevalence of illness anxiety disorders among adults in Taif City, Saudi Arabia

Approximately 105 (25.3%) exhibited anxiety, as depicted in Figure 1. Among the anxious participants, 3 (2.9%) were diagnosed with cardiac diseases such as congenital heart disease, cardiomyopathy, and coronary artery disease. Furthermore, 28 (27%) of the anxious individuals were hospitalized due to cardiac symptoms like chest pain, palpitations, and shortness of breath. Roughly 26 (25%) of anxious participants exhibited normal examination results after hospitalization, with no evidence of cardiac disease (Table 2).

**Table 2:** Clinical characteristics of the anxious participants *n (%)

Characteristic	N = 105^*^
Have you been diagnosed with cardiac diseases?	
No	102 (97%)
Yes	3 (2.9%)
If yes, what was your diagnosis?	
Cardiomyopathy	1 (1.0%)
Congenital heart disease	1 (1.0%)
Coronary artery disease	1 (1.0%)
Rheumatic heart disease	0 (0%)
If yes, Did you receive treatment?	
No	2 (1.9%)
Yes	1 (1.0%)
Have you ever had cardiac symptoms that made you go to the hospital?	
No	77 (73%)
Yes	28 (27%)
If yes, what was it?	
chest pain	10 (10%)
palpitation	9 (8.6%)
Shortness of breath	9 (8.6%)
swelling of the feet, ankles, legs	0 (0%)
If yes, What was the result of your visit to the hospital?	
I was diagnosed with heart disease	2 (1.9%)
The medical examinations were normal, and I was not diagnosed with heart disease	26 (25%)

Females exhibited a higher mean IAD than males (p=0.006). Additionally, younger participants demonstrated a higher mean IAD than their older counterparts (p=0.006). Singles had a higher mean IAD than married and divorced individuals (p=0.012). Participants with a history of hospitalization due to heart symptoms displayed a higher mean IAD compared to those without such a history (p=0.003). Moreover, participants hospitalized due to chest pain and shortness of breath exhibited a higher mean IAD than those with symptoms like palpitations and swelling of the feet, ankles, and legs (p=0.001) (Table 3). 

**Table 3:** Association between illness anxiety disorder and demographic characteristics of the participants ^1^Mean±SD; ^2^One-way ANOVA; Welch Two Sample t-test

Characteristic	N = 415^1^	p-value^2^
gender		0.006
Female	14±8	
Male	12±8	
Age		0.006
18-24	14±8	
25-34	13±9	
35-44	12±7	
45-55	10±7	
more than 55	9±6	
Marital status		0.012
Single	14±8	
Married	12±8	
Divorced	11±4	
Education level		0.13
Primary school	13±7	
High school	13±7	
Diploma	8±7	
Bachelor’s	14±8	
Post-graduate	10±5	
Occupation		0.060
Health care provider ( government or private)	14±9	
Government employee	11±8	
Private sector employee	11±5	
Private job	13±8	
Student	14±8	
I’m not working right now	12±8	
income		0.4
Less than 10000 riyals	14±8	
10000-15000 riyals	13±8	
More than 15000 riyals	12±8	
Have you been diagnosed with cardiac diseases?		>0.9
No	13±8	
Yes	13±6	
If yes, what was your diagnosis?		>0.9
Cardiomyopathy	13±5	
Congenital heart disease	10±9	
Coronary artery disease	16±5	
Rheumatic heart disease	15±0	
Have you ever had cardiac symptoms that made you go to the hospital?		0.003
No	13±8	
Yes	15±9	
If yes, what was it?		0.001
chest pain	17±11	
palpitation	15±6	
Shortness of breath	17±9	
swelling of the feet, ankles, legs	6±4	

Post hoc comparisons using the t-test with Bonferroni correction indicated that individuals aged 18-25 years (Mean = 14, SD = 8) had significantly higher mean scores of IAD than those aged 45-55 years (M = 10, SD = 7) (p = 0.007, d = 0.52). The observed effect is moderate, according to Cohen J, 1992 [18]. The test also indicated that singles (Mean = 14, SD = 8) had significantly higher mean IAD than married individuals (Mean = 12, SD = 8) (p = 0.01, d = 0.313). The observed effect is small, according to Cohen J, 1992 [18] (Table 4).

**Table 4:** Post hoc analysis The adjusted p-value is based on Bonferroni correction. The magnitude of effect size is based on Cohen J, 1992 [18].

	Group1	Group2	p-value	Adjusted p-value	effect size	Magnitude of effect size
Age	18-24	25-34	0.353	1	0.277	small
	18-24	35-44	0.226	1	0.137	negligible
	25-34	35-44	0.758	1	0.227	small
	18-24	45-55	0.001	0.007	0.52	moderate
	25-34	45-55	0.079	0.794	0.733	moderate
	35-44	45-55	0.193	1	0.068	negligible
	18-24	more than 55	0.059	0.592	0.347	small
	25-34	more than 55	0.165	1	0.545	moderate
	35-44	more than 55	0.236	1	0.322	small
	45-55	more than 55	0.618	1	0.553	moderate
Gender	Female	Male	0.006	0.006	0.227	small
Marital status	Divorced	Married	0.941	1	-0.049	negligible
	Divorced	Single	0.49	1	-0.43	small
	Married	Single	0.003	0.01	-0.313	small
Have you ever had cardiac symptoms make you go to the hospital?	No	Yes	0.003	0.003	-0.345	small
if yes, what was it?	chest pain	My answer was No	0.034	0.343	0.486	small
	chest pain	palpitation	0.321	1	0.255	small
	chest pain	Shortness of breath	0.907	1	0.034	negligible
	chest pain	swelling of the feet, ankles, legs	0.006	0.058	1.319	large
	My answer was No	palpitation	0.037	0.374	-0.346	small
	My answer was No	Shortness of breath	0.048	0.48	-0.54	moderate
	My answer was No	swelling of the feet, ankles, legs	0.084	0.835	1.09	large
	palpitation	Shortness of breath	0.387	1	-0.268	small
	palpitation	swelling of the feet, ankles, legs	0.03	0.298	1.773	large
	Shortness of breath	swelling of the feet, ankles, legs	0.007	0.074	1.649	large

Married participants had a lower risk of IAD compared to singles (OR: -2.2, 95% CI: -3.9, -0.48), while participants with a history of hospitalization due to heart symptoms had a higher risk of IAD in comparison to those without such history (OR: 2.8, 95% CI: 0.94, 4.7) (Table 5).

**Table 5:** Predictors of illness anxiety disorder ^1^CI = Confidence Interval

Characteristic	Beta	95% CI^1^	p-value
Gender			
Female	—	—	
Male	-1.6	-3.2, 0.05	0.057
Marital status			
Single	—	—	
Divorced	-3.2	-11, 4.6	0.4
Married	-2.2	-3.9, -0.48	0.012
Income			
Less than 10000 riyals	—	—	
10000-15000 riyals	0.44	-1.5, 2.4	0.6
More than 16000 riyals	-0.18	-2.0, 1.7	0.9
Have you ever had cardiac symptoms that made you go to the hospital?			
No	—	—	
Yes	2.8	0.94, 4.7	0.003

## Discussion

This cross-sectional study aimed to determine the prevalence of illness anxiety disorder and its cardiac manifestations. From the 415 participants, the majority of our study participants were females (60%), the group 18-24 (64%), and singles (67%). The vast majority were students (60%) with monthly incomes of less than 10,000 riyals (40%). Most study participants (97%) were never diagnosed with cardiac disease and never had cardiac symptoms (73%).

There might exist some confusion regarding terminology, as the condition referred to as illness anxiety disorder (IAD) in DSM-5 was formerly identified as hypochondriasis. In DSM-5, it's noteworthy that IAD is not considered a diagnosis of exclusion; instead, positive symptoms can be diagnosed independently. This clarification ensures that individuals experiencing symptoms indicative of illness anxiety disorder can receive appropriate recognition and treatment without needing to exclude other potential diagnoses [19,20].

The prevalence of IAD in our study is 25.3%, higher than most of the relevant literature. A review article reported the prevalence across three decades, according to DSM-4 to be about 0.8 and 4.5%. This study found that patients with IAD are reported to have higher rates of anxiety and depression. Nevertheless, different studies reported the overall prevalence in the general population to be up to 13% [21-23]. 

In India, the prevalence of IAD in a sample of 400 attendees at medical outpatient clinics was 7% [8], and 19.8% reported prevalence in different specialty clinics in London [24]. Nevertheless, a German study reported a 0.4% point prevalence of hypochondriasis based on DSM-5 criteria [25]. Another study in Saudi Arabia reported IAD among medical students. The prevalence was 17%; students younger and those with physician visits during the last six months were more likely to report symptoms of IAD [26]. However, Almalki and his colleagues reported a case of IAD that abuses the health care supplements in Saudi Arabia and costs more than $170,000 due to his concerns about having cancer. Luckily enough, after many years of extensive investigations and expensive imaging, the family physician directed him to the psychiatry department and the patient improved after cognitive behavioral therapy [14].

In this study, females were more likely to experience symptoms of IAD than males (p=0.006), younger participants more than older (p=0.006), singles more than married (p=0.01), and those with hospitalization due to cardiac symptoms more than those with no such history (p= 0.003). These results are comparable to findings by Bleichhardt and Hiller, who reported a positive effect of female gender and older age on health anxiety reporting [25]. However, another study reported the associated risk factors of IAD to be a positive family history of hypochondriasis (18%), a history of child abuse (32.1%), and psychiatric comorbidities (71.4%) [8].

Upon assessment of the clinical characteristics of anxious participants, only (2.9%) were diagnosed with cardiac diseases, which include cardiomyopathy, congenital heart disease, and coronary artery disease. The most reported cardiac symptoms were chest pain (10%), palpitations (8.6%), and shortness of breath (8.6%). It is suggested that psychiatric disorders (like anxiety disorders) can be associated with an increased incidence of medical illnesses. A case-control study reported a higher hazard of cardiovascular diseases among patients with anxiety disorders (hazard ratio 2, 95% CI 1.09-3.65) [27]. On the other hand, people with different physical illnesses can experience a higher frequency of health anxiety. A study was conducted by Tyrer et al. to determine the prevalence of health anxiety (hypochondriasis) in different healthcare clinics. The highest prevalence was found among neurology clinic attendees (24.7%), followed by pulmonology clinics (20.9%), gastroenterology clinics (19.5%), then cardiology and endocrinology clinics (19.1 and 17.5%), respectively [24]. Limitations included limited male responses, lack of a control group, and the study's observational nature. Despite these limitations, the study's substantial sample size mitigated the expected biases.

This study highlights the significant influence of health anxiety on physical well-being. We recommend conducting long-term studies to understand the lasting effects of IAD on physical health outcomes. Public health campaigns to increase awareness of IAD, its prevalence, and its potential impact on cardiac health are recommended, which can minimize stigma and promote early intervention. Healthcare provider training to identify and address IAD effectively is crucial and essential for the timely recognition and proper management of individuals experiencing health anxiety.

## Conclusions

This study revealed a significant 25.3% prevalence of IAD among the Saudi population. Given its substantial impact on individuals and the healthcare system, increased attention to this area is essential. Healthcare providers must recognize these disorders to prevent unnecessary investigations and treatments, redirecting patients to psychiatry for more cost-effective and beneficial interventions. Additionally, it is imperative to launch public health initiatives focused on raising awareness about IAD, its prevalence, and its potential implications for cardiac health. These campaigns can play a pivotal role in mitigating the stigma associated with the condition and fostering early intervention efforts. Additionally, it would be imperative to provide comprehensive training to healthcare providers to adeptly recognize and address IAD, thus facilitating timely identification and appropriate management of individuals grappling with health-related anxiety. We recommend future studies be conducted among different cities in Saudi Arabia to evaluate the prevalence of IAD among the Saudi population, and it is better to focus on one age group.

## References

[1] Scarella TM, Boland RJ, Barsky AJ (2019). Illness anxiety disorder: psychopathology, epidemiology, clinical characteristics, and treatment. Psychosom Med.

[2] American Psychiatric Association (2024). Diagnostic and Statistical Manual of Mental Disorders (DSM-5-TR). https://www.psychiatry.org/psychiatrists/practice/dsm.

[3] French JH, Hameed S (2022). Illness Anxiety Disorder. StatPearls [Internet] Treasure Island.

[4] Craske MG, Stein MB (2016). Anxiety. Lancet.

[5] Lemstra M, Neudorf C, D'Arcy C, Kunst A, Warren LM, Bennett NR (2008). A systematic review of depressed mood and anxiety by SES in youth aged 10-15 years. Can J Public Health.

[6] Smith RC (1985). A clinical approach to the somatizing patient. J Fam Pract.

[7] Newby JM, Hobbs MJ, Mahoney AE, Wong SK, Andrews G (2017). DSM-5 illness anxiety disorder and somatic symptom disorder: comorbidity, correlates, and overlap with DSM-IV hypochondriasis. J Psychosom Res.

[8] Pandey S, Parikh M, Brahmbhatt M, Vankar G Clinical study of illness anxiety disorder in medical outpatients. Arch Psyc Psychotherapy.

[9] Kasalova P, Prasko J, Holubova M, Vrbova K, Zmeskalova D, Slepecky M, Grambal A (2018). Anxiety disorders and marital satisfaction. Neuro Endocrinol Lett.

[10] Espiridion ED, Fuchs A, Oladunjoye AO (2021). Illness anxiety disorder: a case report and brief review of the literature. Cureus.

[11] Nash-Wright J (2011). Dealing with anxiety disorders in the workplace: importance of early intervention when anxiety leads to absence from work. Prof Case Manag.

[12] Juruena MF, Eror F, Cleare AJ, Young AH (2020). The role of early life stress in the HPA axis and anxiety. Adv Exp Med Biol.

[13] Todaro JF, Shen BJ, Raffa SD, Tilkemeier PL, Niaura R (2007). Prevalence of anxiety disorders in men and women with established coronary heart disease. J Cardiopulm Rehabil Prev.

[14] Almalki M, Al-Tawayjri I, Al-Anazi A, Mahmoud S, Al-Mohrej A (2016). A recommendation for the management of illness anxiety disorder patients abusing the healthcare system. Case Rep Psychiatry.

[15] Salkovskis PM, Rimes KA, Warwick HM, Clark DM (2002). The Health Anxiety Inventory: development and validation of scales for the measurement of health anxiety and hypochondriasis. Psychol Med.

[16] Abramowitz JS, Deacon BJ, Valentiner DP (2007). The short health anxiety inventory: psychometric properties and construct validity in a non-clinical sample. Cognit Ther Res.

[17] Singh K, Brown RJ (2014). Health-related internet habits and health anxiety in university students. Anxiety Stress Coping.

[18] Cohen J A power primer. Psychol Bull.

[19] Chappell AS (2018). Toward a lifestyle medicine approach to illness anxiety disorder (formerly hypochondriasis). Am J Lifestyle Med.

[20] Stefan S, Zorila A, Brie E (2020). General threat and health-related attention biases in illness anxiety disorder. A brief research report. Cogn Emot.

[21] Magariños M, Zafar U, Nissenson K, Blanco C (2002). Epidemiology and treatment of hypochondriasis. CNS Drugs.

[22] Scarella TM, Boland RJ, Barsky AJ (2019). Illness anxiety disorder: psychopathology, epidemiology, clinical characteristics, and treatment. Psychosom Med.

[23] Weck F, Richtberg S, Neng J (2014). Epidemiology of hypochondriasis and health anxiety: comparison of different diagnostic criteria. Curr Psychiatry Rev.

[24] Tyrer P, Cooper S, Crawford M (2011). Prevalence of health anxiety problems in medical clinics. J Psychosom Res.

[25] Bleichhardt G, Hiller W (2007). Hypochondriasis and health anxiety in the German population. Br J Health Psychol.

[26] Ezmeirlly HA, Farahat FM (2019). Illness anxiety disorder and perception of disease and distress among medical students in western Saudi Arabia. Saudi Med J.

[27] Bowen RC, Senthilselvan A, Barale A Physical illness as an outcome of chronic anxiety disorders. Can J Psychiatry.

